# Effect of Physical Exercise on Bone Density and Remodeling in Egyptian Type 1 Diabetic Osteopenic Adolescents

**DOI:** 10.1186/1758-5996-3-25

**Published:** 2011-09-30

**Authors:** Safinaz A Elhabashy, Omaima Mohamed Said, Mervat Harvi Agaiby, Amr A Abdelrazek, Sayed Abdelhamid

**Affiliations:** 1 Pediatrics Department, Faculty of Medicine, Ain Shams University, Cairo, Egypt; 2 Pediatrics Department, National Research Center, Cairo, Egypt; 3 Department of Medical Biochemistry, National Research Center, Cairo, Egypt

## Abstract

**Background:**

The study was planned to assess effect of physical exercise on bone remodeling in type I diabetics with osteopenia.

**Methods:**

Twenty-four type I diabetes mellitus (DM1) with osteopenia (10 females and 14 males) were compared to thirty-eight age- and sex-matched healthy control individuals (20 females and 18 males) for biochemical and radiologic parameters of bone mass. Laboratory investigations included serum and urinary calcium, inorganic phosphorus, alkaline phosphatase, and serum "procollagen type 1 N-terminal propeptide (P1NP). Bone densitometry was assessed at neck femur using Dual Energy X-ray Absorptiometry (DEXA). Serum P1NP and DEXA were reevaluated after a planned exercise program.

**Results:**

Patients and controls were comparable with respect to serum as well as urinary biochemical parameters of bone mass namely; calcium, phosphorus and total serum alkaline phosphatase. Osteopenic DM1 patients displayed lower mean serum P1NP than control group (20.11 ± 6.72 ug\dL versus 64.96 ± 34.89 ug\dL; p < 0.05). A significant correlation was observed between BMD and degree of glycemic control reflected by serum glycated hemoglobin (r = -0.44, p, 0.030). Bone densitometry correlated with serum P1NP (r = -0.508, p, 0.011). After a planned regular exercise for 3 months, serum P1NP and BMD levels increased with percentage change of 40.88 ± 31.73 and 3.36 ± 2.94, respectively. Five patients resumed normal densitometry and they were all males.

**Conclusion:**

Diabetic osteopenic patients displayed lower serum levels of procollagen type 1 N-terminal propeptide which reflects poor bone formation. A 3-months planned exercise program was associated with improvement of bone densitometry and significant increment of serum P1NP.

## Background

Osteopenia is not uncommon in children and adolescents with DM1 [1]. The mechanism by which the bone loss occurs in diabetic patients could be explained by a reduction of insulin\insulin like growth factor 1 action, sustained hyperglycemic state, generation of glycosylation end--products, and diabetic complications such as neuropathy, nephropathy, and retinopathy. Osteoblast deficit is suggested to play important role in the occurrence of diabetic osteopenia [2]. Bone formation at onset of DM1 is not impaired. The introduction of insulin therapy together with the achievement of a good metabolic control determine an increase of bone matrix formation coupled with a decrease of bone resorption, which determine a positive balance of bone remodeling [3]. Giannini et al. [4] stated that exercise seems to be very effective in reducing the side effects of the disease, providing three major benefits for exercise; it reduces the diabetic needs for insulin, reduces platelets adhesiveness, and reduces severity of risk factors of coronary artery disease (hypertension, obesity, blood lipids, and serum uric acid). Special considerations are needed for physically active individuals with type 1 diabetes mellitus. Although regular activity is beneficial for all patients, vigorous exercise can cause major disturbances in blood glucose. The glycemic response depends largely on the type, intensity and duration of the activity, circulating insulin and glucose counter regulatory hormone concentrations, type and timing of food, metabolic control, muscle mass/number of muscles used in activity, conditioning, and Degree of stress/competition involved in the activity and timing of activity [5-7]. The current prospective study was planned to assess bone remodeling status in osteopenic DM1 patients and its relation to diabetes duration and glycemic control, and the effect of planned physical exercise on bone mass.

## Materials and methods

### Subjects

We studied 24 pubertal and adolescent patients with DM1; 10 females and 14 males whose ages' range was 14-20 years. They were regularly attending Outpatients' Diabetes Specialized Clinic, of Children's Hospital, Ain Shams University, Cairo, Egypt. Patients were compared to age- (14-20 years) and gender-matched (20 females and 18 males) healthy control subjects. Exclusion criteria were presence of diabetic complications, celiac disease, liver disease, presence of any associated musculoskeletal or metabolic bone diseases, any chronic illness other than diabetes, any medications other than insulin, and delayed puberty. The study plan has been approved by local ethical committee with a federal number: FWA00006444.

### Methods

All patients were subjected to full history taking including age at onset of the disease, duration of diabetes, presence of diabetic complications, presence of any musculoskeletal disease and history about regular exercise or medications other than insulin. Full clinical examination was performed with special stress on growth parameters (weight, height, and pubertal stage), blood pressure measurement and fundus examination.

#### Laboratory investigation

Fasting morning blood samples were drawn for determinations of biochemical parameters. Serum and urinary levels of calcium, inorganic phosphorous and serum total alkaline phosphatase were measured by standard laboratory techniques on Hitachi 747. Plasma HbA1c was determined by an ion-exchange high performance liquid chromatography. Nephropathy was excluded by measuring microalbuminurea in relation to creatinine clearance (Synchrony CX5 system) on 2 occasions one month apart. Serum procollagen type 1 N-terminal propeptide (P1NP) was estimated using rapid equilibrium radioimmunoassay [8] (commercial antisera specifically directed against the amino-terminal propeptide supplied by Orion Diagnostica, Espoo, Finland) before and after exercise program.

##### Bone densitometry

Bone mineral density at left neck of femur was measured by dual-energy X-ray absorptiometry (DEXA) using Hologic QDR 1000/W scanner, Waltham, MA, USA) at study entry and repeated after an exercise program. Results are expressed as BMD (g/cm2). Low BMD was defined according to T-scores, calculated on the basis of the normal reference values. The T scores are reported as the number of standard deviations below the young adult mean (normal, > -1; osteopenia, -1 to -2.49; osteoporosis, -2.5) [9].

#### Exercise program

Diabetic patients were offered an aerobic exercise program in diabetes specialized clinic, 3 times a week for 3 months. The schedule was as follows: 15 minutes warming up for abdominal and back muscles, five minutes rest, then 20 minutes on ergo meter, ten minutes rest and lastly 20 minutes on ergo meter with constant speed and resistance. Eleven osteopenic DM1 patients were able to regularly complete such exercise regimen.

#### Precautions to avoid hypoglycemia

For 2 weeks prior study, patients were prepared as follows to join exercise program; 1: exercise was offered at least 4 hours from rapid acting insulin dose with a blood glucose level above 150 mg/dl just prior exercise; 2: glucose monitoring immediately after, 2, 6, and 12, 24 and 36 hours after exercise; 3: Adequate carbohydrate replacement before, during, and after exercise (average 15-30 gm) if blood sugar is less than 100 mg/dl; and 4: reduction of pre-prandial rapid-acting insulin doses (10-25%).

### Statistical analysis

This was performed with SPSS statistical software, version 15. Statistical significance of difference between variables was analyzed by student "t", Mann-Whitney, and Wilcoxon rank, and paired "t" tests. Relation between variables was studied by correlation coefficient "r" test. Percentage change in serum P1NP and BMD post exercise was calculated (value after minus value before, divided by value before, times 100). Values of p < 0.05 were considered significant. All statistical tests were two-tailed.

## Results

### Clinical parameters

Patients with DM1 were comparable to control subjects for mean age (Z = 0.33, p = 0.742) gender distribution (X^2^= 0.708, p = 0.400), weight percentile (Z = 0.786, p = 0.432), and height percentile (Z = 0.546, p = 0.585) as shown in table 1.

**Table 1:** Clinical and Biochemical parameters in diabetic patients and control subjects.

Clinical parameters	Controls (n = 38)	Patients (n = 24)
Age (yr)	16.97 ± 1.88 (14-20)	17.17 ± 2.01 (14 - 20)
Duration (yr)	----------------------	7.5 ± 2.7 (4-14)
Weight percentile	30.43 ± 22.28 (3 - 60)	41.42 ± 28.17 (3-77)
Height percentile	31.43 ± 26.59 (3-75)	27.46 ± 26.51 (3-90)
**Biochemical markers**		
Serum calcium (mg/dl)	9 ± 0.22 (8.8 - 9.4)	9.12 ± 0.19 (8.8 - 9.5)
Serum phosphorus (mg/dl)	3.7 ± 0.19 (3.5 - 4)	3.75 ± 0.28 (3.1 - 4.5)
Serum alkaline phosphatase (U/L)	190.86 ± 44.72 (153 - 273)	245.9 ± 137.23 (115 - 803)
Urinary calcium (mg/g creatinine)	181 ± 31.9 (132 - 222)	187.75 ± 38.9 (112 - 266)
Urinary phosphorus (mg/g creatinine)	680.9 ± 216.1 (375 - 943)	721.4 ± 179 (369 - 985)
Serum P1NP (ug/dl)	64.96 ± 34.89 (21.9 - 166.6)	20.11 ± 6.72 (11.5 - 33.9)*
**Bone densitometry**		
Bone mineral density (g/cm2)	0.98 ± 0.12 (0.86-1.12)	0.87 ± 0.10 (0.70-1.13)*

### Biochemical markers

As for biochemical markers of bone remodeling, mean levels of serum and urinary calcium, phosphorus and alkaline phosphatase in DM1 patients were not significantly different from control subjects. However, patients displayed lower mean serum P1NP when compared with controls, p = < 0.001 (table 1). A non significant difference was noted in mean serum P1NP between boys and girls whether in patients or controls (p = 0.051 and 0.09, respectively, data not shown). No correlation was observed between serum P1NP and degree of glycemic control as assessed by mean of last 4 glycated hemoglobin concentrations (HbA1c) (r = -0.28, p = 0.18) as illustrated in table 2.

**Table 2:** Correlation of serum P1NP and BMD before exercise in diabetic patients.

	Serum P1NP	Bone mineral Density
Diabetes duration	r = 0.244	r = -0.283
	p = 0.25	p = 0.18
Weight percentile	r = 0.231	r = 0.452
	p = 0.182	p = 0.021*
Glycated hemoglobin	r = -0.28	r = 0.444
	p = 0.175	p = 0.03*
Bone mineral density	r = 0.508	
	p = 0.011*	

*Significant

### Bone densitometry

**Figure 1:**
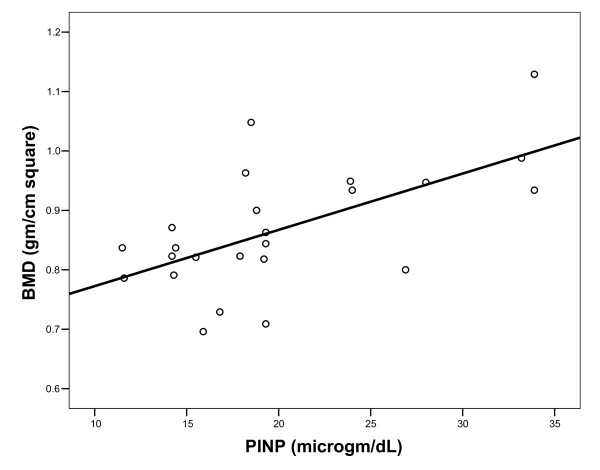
**Correlation between serum P1NP and BMD (r = 0.508; p = 0.011)**.

All included patients were osteopenic, hence, their mean BMD was lower than age and sex matched healthy subjects (p = 0.026). An inverse correlation was observed between bone densitometry and glycated hemoglobin (table 2). BMD correlated with serum P1NP (Figure 1).

### Effect of exercise on bone mass

**Figure 2:**
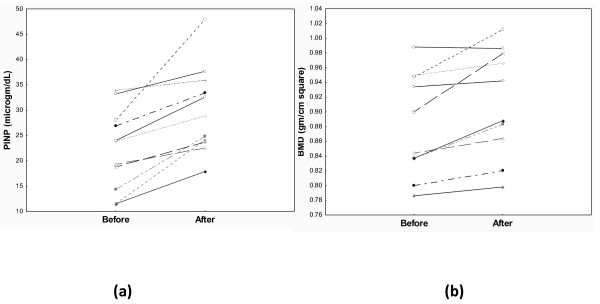
**Effect of exercise on individual values of BMD (a) and serum P1NP (b)**.

Eleven patients only (seven males) were able to complete 3-months regular exercise and were eligible for evaluation. It is noteworthy mentioning that none of DM1 patients experienced severe or symptomatic hypoglycemic attack during or within 24 hours post exercise. Table 3 shows the effect of exercise on bone densitometry and serum P1NP in diabetic patients. With respect to BMD, mean values of BMD rose to a significant level after exercise and 5 male patients turned non osteopenic. Serum P1NP levels increased to levels still significantly lower than control values (Figure 2).

**Table 3:** Effect of exercise on bone mass in eleven diabetic patients

	Before	After	Percentage Change	p
**Serum P1NP** (ug/dl)	22.32 ± 7.92 (11.5 - 33.9)	29.95 ± 8.59* (17.9- 47.9)	40.88 ± 31.73 (5.9 ± 107.76)	0.003*
**BMD **g/cm2	0.89 ± 0.07 (0.79-0.99)	0.92 ± 0.07* (0.80-1.01)	3.36 ± 2.94 (-0.2-8.78)	0.004*

*Significant

## Discussion

It is demonstrated that several years after the diagnosis of clinical diabetes mellitus, children have decreased lumbar bone mineral density compared to control group or healthy children matched for age, height, sex and pubertal status. This decrease in BMD was already present in the patients less than two years after diagnosis of DM1 suggesting that a defect in bone mass accretion occurs quite early in the disease [10]. Twenty four pubertal and adolescent diabetics with low BMD were recruited before starting any drug therapy other than insulin. BMD was measured at the femoral neck, as this site has the greatest value in predicting both hip and other osteoporotic fractures [11]. Biochemical measurement of bone turnover was assessed in a trial to explain osteopenia. Alterations of calcium and Vitamin D3 balance have also been implicated as contributing factors of diabetic osteopenia by some authors. In the current cohort, serum and urinary calcium and phosphorus concentrations were found to be normal in all patients and mean levels were comparable to age- and sex-matched control subjects, similar to findings reported by others [12]. Serum total alkaline phosphatase (AP) is the most widely used marker of bone metabolism due to the wide availability of inexpensive and simple methods. Once liver disease is ruled out, serum levels of total AP provide a good impression of the extent of new bone formation and osteoblast activity [13,14]. We found that serum levels of total AP were comparable to control subjects. However, mean serum P1NP, the other marker of new bone formation, was lower in our DM1 osteopenic patients. Aminoterminal propeptides of procollagen 1 (P1NP) being generated from newly synthesized collagen, are considered quantitative measures of newly formed type 1 collagen, the most abundant form of collagen found in bone. Although type I collagen propeptides may also arise from other sources, most of the non-skeletal tissues exhibit a slower turnover than bone, and contribute very little to the circulating propeptide pool [15]. Reduced osteoblast activity has been investigated extensively in DM1 patients with osteopenia [16]. So, the findings of low serum levels of P1NP in the current work may be considered while explaining causes of osteopenia in DM1.

Consequences of poor metabolic control and/or insulin deficiency have been reported to account for increased bone resorption and bone loss. Presence of correlation between BMD and glycated hemoglobin in the current study is in line with others' finding of poor metabolic control in diabetics with consequent bone loss. The expected negative correlation between BMD and diabetes duration that has been reported by others[17,18] wan not proven in our cohort.

In the current study, 11 patients were able to complete a planed exercise program for 3 months. Mechanical loading provided is known to be an anabolic stimulus for bone as mechanic-sensing apparatus in bone directs osteogenesis to where it is most needed for improving bone strength [19]. The most easily demonstrated interaction between physical activities and bone mass is the substantial bone loss that follows complete immobilization. When designing an exercise program for patients with osteoporosis, it should not be harmful in terms of hypoglycemia, worsening complications, and fractures. Precautions were taken to avoid symptomatic hypoglycemia and were effective in our patients as none reported any episode. When they were re-evaluated post exercise, BMD improved in patients with a variable degree and 5 out of 11 (45.5%) resumed normal BMD for age, sex and height. This might be explained in part by new bone formation [20] reflected by significant increase in serum P1NP. Our finding is supported by the study of Burke et al. [21]. The latter assessed the relationship between exercise, diabetes, and bone metabolism, and found that there is an alteration in the mineral content of the femurs in diabetic animals than controls, and that mineral content of diabetic animals placed on exercise regimen revealed values closer to controls' levels.

In conclusion, DM1 osteopenic patients displayed lower serum P1NP levels which reflect poor bone formation. Meanwhile, biochemical markers of bone mass reflected by serum and urinary values of calcium, phosphorus and serum alkaline phosphatase were all within normal range. A 3-months planned exercise program was associated with improvement of bone densitometry and significant increment in serum P1NP.

## Abbreviations

DEXA: dual energy X-ray absorptiometry; P1NP: procollagen type 1 N-terminal propeptide. BMD: bone mineral density; BMI: body mass index; NPH: neutral protamine Hagedorn; HbA1c: glycosylated hemoglobin concentrations; DM: diabetes mellitus; AP: alkaline phosphatase.

## Competing interests

The authors declare that they have no competing interests.

## Authors' contributions

SAE designed the study, supervised the work and drafted the manuscript. OMS and AAA shared in patients' follow up, and data collection. MHA carried out the laboratory studies, analyzed the data and reviewed the manuscript. SAA conducted practical work, collected data. All authors approved the manuscript.
